# Supplementary material for: The Small-Molecule Wnt Inhibitor ICG-001 Efficiently Inhibits Colorectal Cancer Stemness and Metastasis by Suppressing MEIS1 Expression
Source: Int J Mol Sci. 2021 Dec 14;22(24):13413. doi: 10.3390/ijms222413413 (PMC8704261; doi:10.3390/ijms222413413)
Supplement: Supplementary file 1 [file ijms-22-13413-s001.zip › ijms-1478489-supplementary.pdf]

Supplementary Materials for

The small molecule Wnt inhibitor ICG-001 efficiently inhibits colorectal cancer stemness and metastasis by suppressing MEIS1 expression

Jang-Hyun Choi[1,*], Tae-Young Jang[1,*], So-El Jeon[1,*], Jee-Heun Kim[1], Choong-Jae Lee[1], , Hyeon-ji Yun[1], Ji-Youn Jung[2], So-Yeon Park[1,3,†], Jeong-Seok Nam[1,3,†]

[1]School of Life Sciences, Gwangju Institute of Science and Technology, Gwangju, 61005, Republic of Korea

[2]Department of Companion and Laboratory Animal Science, Kongju National University, Yesan, Chungcheongnam 32439, Republic of Korea

[3]Cell Logistics Research Center, Gwangju Institute of Science and Technology, Gwangju, 61005, Republic of Korea

* Equally contributed

† Correspondence: namje@gist.ac.kr (J.-S.N.); sypark0125@gist.ac.kr (S.-Y.P.); Tel.: +82-62-715-2893 (J.-S.N.)

1. Supplementary Materials and Methods

1.1. Western blot analysis

Protein isolation was conducted, as previously described [8]. The blot was probed with primary antibodies against LEF1 (#2230, Cell Signaling Technology, Boston, MA, USA), CTNNB1 (#9562, Cell Signaling Technology), and MEIS1 (ab229962, Abcam, Cambridge, UK). As a loading control, anti-β-actin antibody (A5316, Sigma Aldrich, Saint Louis, MO, USA) was used. Subsequently, the blots were washed in TBST (10 mmol/l Tris-HCl, 50 mmol/l-NaCl, and 0.25% Tween-20) and incubated using a horseradish peroxidase-conjugated secondary antibody. Target proteins were detected using enhanced chemiluminescence reagents (Thermo-Scientific, Waltham, MA, USA).

1.2. Sphere-forming assay

Sphere-forming assay was performed, as previously described [5]. Briefly, cells were seeded in 6-well plates coated with poly-HEMA in serum-free media supplemented with EGF, FGF, B27, and heparin, and cultured for 7 days for sphere formation. The number of spheres from each replicate well was counted under an inverted microscope at 50× magnification using the Image-Pro Premier 9.0 (Media Cybernetics, Rockville, MD, USA). Tumor sphere-forming efficiency (TSFE) was calculated as the number of spheres divided by the number of seeded cells.

1.3. Real-time reverse transcription-polymerase chain reaction (RT-qPCR)

Total RNA was extracted using RNAiso (Takara, Shiga, Japan), and RNA purity was verified by measuring the 260/280 absorbance ratio. Using PrimeScript™ 1st Strand cDNA Synthesis Kit (Takara), the first strand cDNA was synthesized, and one-tenth of the cDNA was used for each PCR mixture containing Power SYBR® Green PCR Master Mix (Applied Biosystems, Foster City, CA, USA). RT-PCR was conducted

using a StepOnePlus Real-Time PCR System (Applied Biosystems). The PCR primer sequences are listed in Supplementary Table 1.

1.4. Immunofluorescence assay

Immunofluorescence assay was conducted, as previously described [8]. Cells were seeded on poly-L-lysine and collagen I-coated cover glasses and fixed with 4% formalin. Tissue slides and cells were permeabilized using 0.3 M glycine and 0.3% Triton X-100, after which they were blocked with 2% normal swine serum (DAKO). As previously described, staining was conducted using primary anti-MEIS1 (1:200). All nuclei were counterstained with DAPI.

1.5. Animal study

To examine the effect of ICG-001 on self-renewal ability of CRC cells, *in vivo* limiting dilution assay (LDA) was conducted as described in our previous report [9]. Briefly, HCT116 cells were subcutaneously injected into male NSG mice (1×10^6 cells/mouse). On the 7th day after inoculation, when tumor volume attained approximately 100 mm^3, mice were randomly divided into two groups and treated daily with ICG-001 at 100 mg/kg or vehicle control (i.p., 4% ethanol, 8% Tween 80 in PBS, n = 5/group). On the 49th day, mice were sacrificed, and single tumor cells were isolated and injected into male NSG mice in various cellular numbers (1×10^3, 1×10^4, 1×10^5, and 1×10^6/mouse, n = 5/each group) without any additional ICG-001 treatment. On the 70th day after inoculation, the incidence of tumor-bearing mice was determined by necropsy. The LDA plots and statistical significance were calculated using web-based software (http://bioinf.wehi.e.,du.au/software/elda).

To compare the self-renewal ability between wild-type and MEIS1-OE HCT116 cells, in vivo LDA was performed. Briefly, cells were subcutaneously injected into male

NSG mice in various cellular numbers (1×10^3, 1×10^4, 1×10^5, and 1×10^6/mouse, n = 5/each group). On the 21st day after inoculation, the incidence of tumor-bearing mice was determined by necropsy. The LDA plots were generated as described above.

For the liver metastasis mouse model, HCT116-luc cells (1×10^5 cells/mouse) were inoculated into spleens of male NSG mice, followed by splenectomy, and surviving cells that grew in distant organs then contributed to the formation of liver metastasis. The extent of liver metastasis was monitored weekly via visualizing luciferase activity for 18 days using the IVIS Lumina Imaging System. After sacrifice, livers were removed, formalin-fixed, and paraffin-embedded to determine liver metastasis. To determine the effect of ICG-001 treatment on liver metastasis, male mice were randomly divided into two groups the next day after splenic injection of HCT116-luc cells (1×10^6 cells/mouse) and treated daily with ICG-001 (100 mg/kg) or vehicle control (n = 5/group) until necropsy. The extent of liver metastasis was monitored weekly via visualizing luciferase activity for 21 days as described above.

2. Supplementary Table

2.1. Supplementary Table S1: The list of PCR primer and siRNA sequences

Primer & siRNA Sequence		
**OCT1**	Forward	CCCTGTCTCAGCCCATACAGA
	Reverse	GCTGCAAATTGGTGGTTGGAT
**OCT4**	Forward	CACCTTCCCTCCAACCAGTTGC
	Reverse	GGGCTCTCCCATGCATTCAAAC
**SOX2**	Forward	AAATGGGAGGGGTGCAAAAGAGGAG
	Reverse	CAGCTGTCATTTGCTGTGGGTGATG
**SOX4**	Forward	GGTCTCTAGTTCTTGCACGCTC
	Reverse	CGGAATCGGCACTAAGGAG
**NANOG**	Forward	TGGGATTTACAGGCGTGAGCCAC
	Reverse	AAGCAAAGCCTCCCAATCCCAAAC
**SOX9**	Forward	CATGAGCGAGGTGCACTCC
	Reverse	TCGCTTCAGGTCAGCCTTG
**CMYC**	Forward	CAAGTATACGTGGCAATGCGT
	Reverse	TCAAGAGTCCCAGGGAGAGT
**KLF4**	Forward	ACGATCGTGGCCCCGGAAAAGGACC
	Reverse	CAACAACCGAAAATGCACCAGCCCCAG
**LGR5**	Forward	AATCCCCTGCCCAGTCTC
	Reverse	CCCTTGGGAATGTATGTCAGA
**KLF5**	Forward	CCCTTGCACATACACAATGC
	Reverse	GGATGGAGGTGGGGTTAAAT
**ALDH1A1**	Forward	CTGGTTATGGGCCTACAGCA
	Reverse	GCATTGTCCAAGTCGGCATC
**BMP4**	Forward	GATCTTTACCGGCTTCAGTCTGGG
	Reverse	ACCTCGTTCTCAGGGATGCTGC
**BAMBI**	Forward	CGACAGACATCTGCCAAGCCAAAC
	Reverse	GAAGTCAGCTCCTGCACCTTGGTG
**WNT1**	Forward	CGGCGTTTATCTTCGCTATC
	Reverse	TTCGATGGAACCTTCTGAGC
**WNT3A**	Forward	TGTTGGGCCACAGTATTCCT
	Reverse	GGGCATGATCTCCACGTAGT
**AXIN2**	Forward	GAGACCCCACCGTGTCAGC
	Reverse	AACAGGTAAGCACCGTCTTGAT
**CTNNB1**	Forward	CCTGTATGAGTGGGAACAGGG
	Reverse	TCAGCCAAACGCTGGACATT
**LEF1**	Forward	ACAGCGACCCGTACATGTCAAA
	Reverse	TGGACATGCCTTGCTTGGAGTT
**TCF4**	Forward	GCCTTAGGGACGGACAAAGA
	Reverse	CATAGTTCCTGGACGGGCTT

**CCND1**	Forward	CTGTGCATCTACACCGACAAC
	Reverse	CCTCCGCCTCTGGCATTTT
**APC**	Forward	CTGGCAACATGACTGTCCTT
	Reverse	TGCAAACCTCGCTTTGAAGAA
**CDH1**	Forward	TTTGACGCCGAGAGCTACAC
	Reverse	CACACCATCTGTGCCCACTT
**FXYD5**	Forward	TCCCACTGATGACACCACGA
	Reverse	AAACCAGATGGCTTGAGGGT
**VEGFA**	Forward	GGCCTCCGAAACCATGAACT
	Reverse	GCAGTAGCTGCGCTGATAGA
**MMP2**	Forward	CAATGGCAAGGAGTACAACAGC
	Reverse	TGTCATAGGATGTGCCCTGG
**MMP7**	Forward	GGCCATTCTTTGGGTATGGGA
	Reverse	TGGATGTTCTGCCTGAAGTTT
**KRAS**	Forward	TGGATATTCTCGACACAGCAGG
	Reverse	GGACCATAGGTACATCTTCAGAGTC
**IGF1**	Forward	CCGCAGGATCCTTTGCTCTG
	Reverse	TGCAAGGTGCAAATCACTCC
**CXCR4**	Forward	GAACCCTGTTTCCGTGAAGA
	Reverse	CTTGTCCGTCATGCTTCTCA
**CDKN2A**	Forward	CTGGACACGCTGGTGGTG
	Reverse	AATCGGGGATGTCTGAGGGA
**CD44**	Forward	CTACTGTACACCCCATCCCAG
	Reverse	AGCCATTTGTGTTGTTGTGTGA
**CD133**	Forward	AGAAATGCACCAGCGACAGA
	Reverse	TCAAGAGAGTTCGCAAGTCC
**MEIS1**	Forward	GTTGAAGTAGGAAGGGAGCCAG
	Reverse	GCTGTGTGCGGGTACTGATG
**VIM**	Forward	AACTTAGGGGCGCTCTTGTC
	Reverse	CGCTGCTAGTTCTCAGTGCT
**CDH2**	Forward	TGACCAGCCTCCAACTGGTAT
	Reverse	CAGGGGCTTTGTCACCGA
**SNAI1**	Forward	CTGGGTGCCCTCAAGATGCA
	Reverse	CCGGACATGGCCTTGTAGCA
**SNAI2**	Forward	CACAGTGATGGGGCTGTATG
	Reverse	GCCTCCAAAAAGCCAAACTA
**ZEB1**	Forward	TGGTGATGCTGAAAGAGACG
	Reverse	TGCACTGAGTGTGGAAAAGC
**TJP1**	Forward	GGAGAGGTGTTCCGTGTTGT
	Reverse	GAGCGGACAAATCCTCTCTG
**L1CAM**	Forward	CACTATGGCCTTGTCTGGGA
	Reverse	ACATACTGTGGCGAAAGGGA
**siLEF1**	Sense	GACGGUAACUUGGCUGCAU(dTdT)
	Antisense	AUGCAGCCAAGUUACCGUC(dTdT)

**siCTNNB1**	Sense	ACGACUAGUUCAGUUGCUU(dTdT)
	Antisense	AAGCAACUGAACUAGUCGU(dTdT)
**siMEIS1 #1**	Sense	CAAAAAGCGUGGCAUCUUU(dTdT)
	Antisense	AAAGAUGCCACGCUUUUU(dTdT)
**siMEIS1 #2**	Sense	GACUCAGAAUUACAUCCA(dTdT)
	Antisense	UUGGAUGUAAUUCUGAGUC(dTdT)
**siMEIS1 #3**	Sense	CUGACAUUCAGGCCCAAGU(dTdT)
	Antisense	ACUUGGGCCUGAAUGUCAG(dTdT)
**siMEIS1 #4**	Sense	CUCUACCUAAAUAGUCGAU(dTdT)
	Antisense	AUCGACUAUUUAGGUAGAG(dTdT)

3. Supplementary Figures

3.1. Supplementary Figure S1

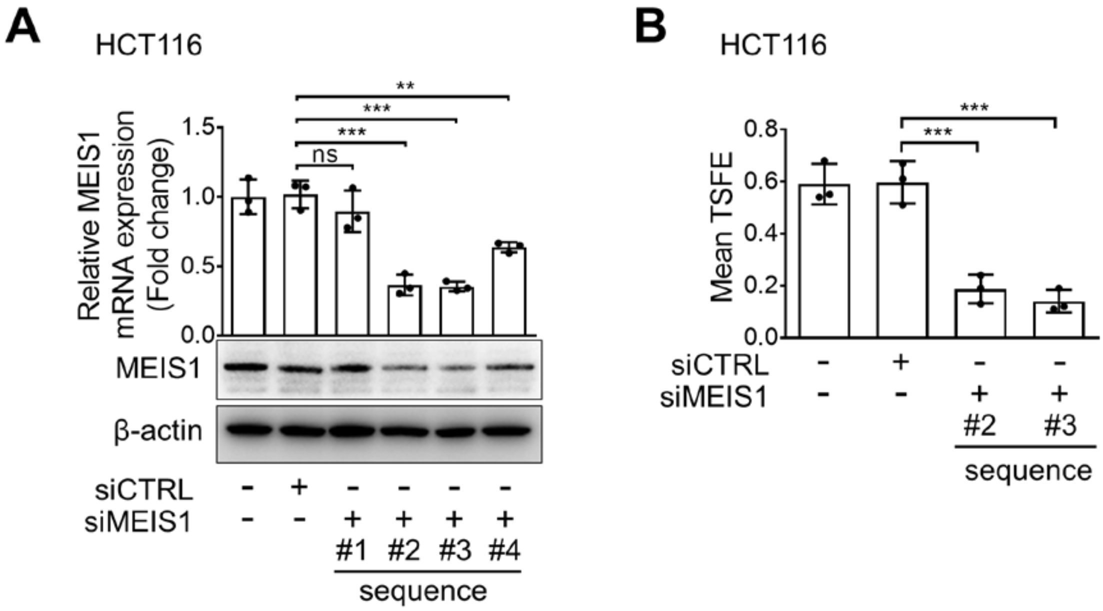

Supplementary Figure S1. MEIS1-knockdown reduces self-renewal ability of CRC cells. (A) RT-qPCR and Western blot analyses conducted 48 hours after MEIS1-knockdown (n = 3/group). (B) Sphere formation assay comparing self-renewal ability between MEIS1-knockdown and control cell s (n = 3/group). Statistical differences were determined using one-way analysis of variance (ANOVA) with Dunnett's multiple comparisons test. **, and *** indicate $p < 0.01$, and $p < 0.001$, respectively.

3.2. Supplementary Figure S2

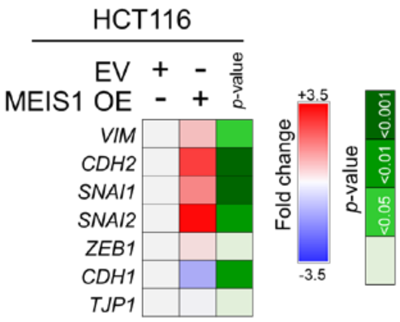

Supplementary Figure S2. MEIS1-overexpression promotes the aggressive phenotypeof CRC cells. RT-qPCR validation confirming the global increases in mesenchymal markers and reductions in epithalial markers by MEIS1-overexpression. The data are presented as a heatmap with fold changes and p-values. Statistical differences were determined using Student's t-test.
